# Smaller baseline subcortical infarct volume predicts good outcomes in patients with a large core in early acute ischemic stroke after endovascular treatment

**DOI:** 10.3389/fnins.2023.1063478

**Published:** 2023-02-06

**Authors:** Yiming Gu, Yasuo Ding, Yu Hang, Yuezhou Cao, Zhenyu Jia, Linbo Zhao, Ying Liu, Sheng Liu

**Affiliations:** ^1^Department of Interventional Radiology, The First Affiliated Hospital of Nanjing Medical University, Nanjing, China; ^2^Department of Interventional Radiology, Suzhou Municipal Hospital Affiliated to Nanjing Medical University, Suzhou, China; ^3^Department of Neurosurgery, Taizhou People’s Hospital, Taizhou, China; ^4^Department of Neurology, Taizhou People’s Hospital, Taizhou, China

**Keywords:** acute ischemic stroke, endovascular thrombectomy, infarct volume, predictor, good outcomes

## Abstract

**Background:**

Patients with acute ischemic stroke (AIS) and a large core may benefit from endovascular treatment (EVT) in the early time window.

**Purpose:**

To examine the prognostic factors for good outcomes in patients with a large core (70–130 ml) after EVT.

**Materials and methods:**

We retrospectively reviewed 40 patients who met the criteria from October 2019 to April 2021. Based on the modified Rankin Score (mRS) at 90 days, the patients were divided into a good outcome group (mRS 0–2) and a poor outcome group (mRS 3–6). Baseline and procedural characteristics were collected for unilateral and multivariate regression analyses to explore the factors that influence good outcomes. In particular, the infarct territories were quantified as subcortical infarct volume (SIV) and cortical infarct volume (CIV).

**Results:**

Of the 40 patients included, good outcomes were observed in 11 (27.5%) patients. Younger age, smaller SIV and larger mismatch volume were noted in the good outcome group than in the poor outcome group (all *P* < 0.05). Multivariate logistic regression analysis showed that only a smaller SIV [odds ratio (OR) 0.801; 95% CI 0.644–0.996; *P* = 0.046] was an independent predictor for good outcomes. The receiver operating characteristic curve indicated a moderate value of SIV for predicting good outcomes, with an area under the receiver operating characteristic curve of 0.735 (95% CI 0.572–0.862; *P* = 0.007).

**Conclusion:**

Subcortical infarct volume was a potential useful predictor of good outcomes in patients with a large core after EVT in the early time window.

## Introduction

Alberta Stroke Program Early CT Score (ASPECTS) assessment is widely used as an established decision tool to support treatment in patients with acute ischemic stroke (AIS) due to large vessel occlusions (LVOs), and computed tomography perfusion (CTP) is not recommended in an early time window (Powers et al., 2019). Patients with a low ASPECTS are often not offered endovascular treatment (EVT), as they are thought to have a poor prognosis. Given the well-known inter-rater reliability, non-volumetric nature and relative insensitivity in the very early phases of ASPECTS, the use of CTP increased gradually in clinical practice. CTP-based infarct volume was associated with outcomes in an extended time window (Demeestere et al., 2018; Nogueira et al., 2018), however, there was insufficient evidence and experience in EVT based on infarct volume in the early time window, especially in patients with a large core.

Although a core > 70 ml was an excluding criterion for EVT in several randomized control trials (Campbell et al., 2015; Rebello et al., 2017; Albers et al., 2018) and was not recommended in the guidelines, patients that underwent EVT showed a better prognosis than those with standard medical therapy in some reports. Previous studies suggested that younger age (Rebello et al., 2017), larger mismatch ratio (Kerleroux et al., 2020; Seners et al., 2021), a moderate upper core limit (120–130 ml) (Yoshimoto et al., 2021) and a short stroke onset to recanalization time (ORT) (Yoshimoto et al., 2021) contributed to good outcomes for patients with a large core after EVT. Recent studies have suggested that infarct regions have an important effect on outcomes (Seyman et al., 2016; Ospel et al., 2021). However, the results in the literature showed significant disagreement in patients with a large core. Particularly, no consensus was achieved regarding the effect of the infarct region on functional outcome in patients with a large core in the early time window. A previous study (Xing et al., 2021) examined the contribution of the ASPECTS of the subcortical and cortical regions on non-contrast CT to the clinical outcome, but the estimates were not as precise. Hence, it remains unclear to what extent the causal relationship between EVT and outcome is explained by the baseline subcortical or cortical infarct region.

We presumed that the CTP-derived subcortical or cortical infarct volume (CIV) mediated the relationship between EVT and functional outcomes in the early time window. To our knowledge, no studies have been reported the effect of EVT on outcomes is caused by subcortical or CIV estimated by CTP within 6 h of stroke onset or time last known well. Thus, the objective of this retrospective study was to investigate the effect of CTP-derived infarct region and region-specific infarct volumes on the clinical outcome in patients with a large core in the early time window.

## Materials and methods

### Patient selection

**FIGURE 1:**
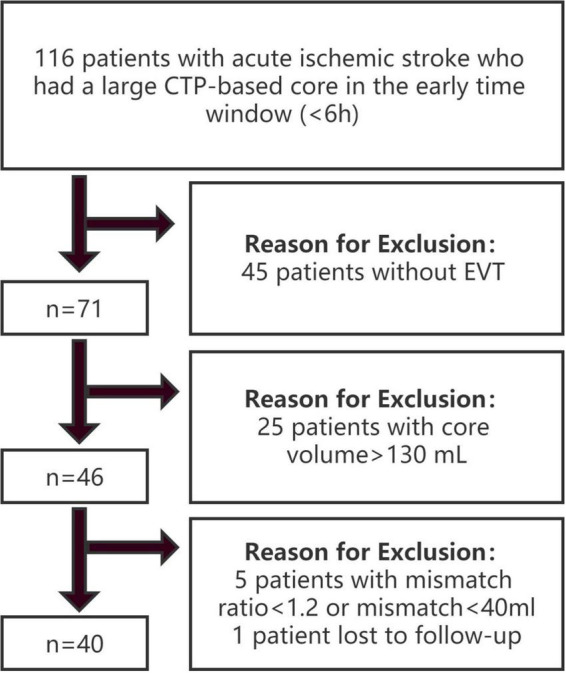
Study flow chart.

Patients in two stroke centers from October 2019 to April 2021 were retrospectively reviewed. The inclusion criteria were as follows: (1) age ≥ 18 years; (2) groin puncture started within 6 h from symptom onset or time last known well; (3) undergoing baseline pretreatment non-contrast CT, CT angiography (CTA), and technically adequate pretreatment CTP; (4) occlusion identified at the internal carotid artery (ICA) or M1 segment of the middle cerebral artery (MCA); (5) ischemic core volume [cerebral blood flow (CBF) < 30%] ≥ 70 ml and ≤ 130 ml; and (6) mismatch ratio ≥ 1.2 (Seners et al., 2021) and absolute mismatch volume ≥ 40 ml (Rebello et al., 2017). The exclusion criteria were as follows: (1) baseline modified Rankin scale (mRS) score ≥ 2; (2) evidence of intracranial hemorrhage on non-contrast CT; and (3) loss to follow-up. The patients were divided into 2 groups based on clinical outcomes at 90 days: the good outcome group with mRS 0–2 and the poor outcome group with mRS 3–6. The study flow chart is shown in Figure 1.

### Image acquisition

**FIGURE 2:**
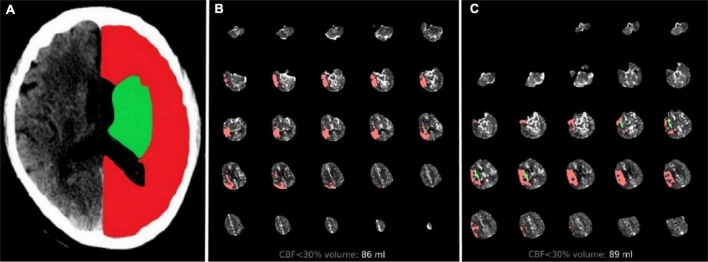
Two cases treated with endovascular treatment taken as example. Illustrative cases: **(A)** Green region represents SIV and red region represents CIV. **(B)** Patient with total core infarct volume = 86 ml, SIV = 1.3 ml, and CIV = 84.7 ml. Them RS score was 1 at 90 days after the follow-up. **(C)** Patient with total core infarct volume = 89 ml, SIV = 9.4 ml, and CIV = 79.6 ml. Them RS score was 4 at 90 days after the follow-up. SIV, subcortical ischemic volume; CIV, cortical ischemic volume; mRS, modified Rankin scale.

Multimodal CT-based imaging, including non-contrast CT, CTP and post-processed series, was conducted at the admission of patients with suspected AIS. The ASPECTS standardized 10-point score on non-contrast CT shows that a score of 10 indicates a normal brain and 1 point is subtracted for each abnormal region. The ischemic core volume [cerebral blood flow (CBF) < 30%] and the mismatch volume based on CTP were calculated by RAPID software. The hypoperfusion intensity ratio (HIR) was defined as the volume of tissue with TMax > 10 s divided by the volume of tissue with TMax > 6 s. Mismatch ratio was defined as Tmax > 6 divided by the core volume. Collaterals were assessed independently of clinical information and assigned a score of 0–4 according to Souza et al. (2012) subcortical infarct volume (SIV) and CIV were manually segmented for all patients with the open-source software ITK-Snap^1^ on RAPID imaging. SIV includes the lesion territory in the caudate, lentiform nucleus, internal capsule, and insular ribbon. The other structures affected were considered CIV (Figure 2A).

### Endovascular treatment

All procedures were performed under local anesthesia/conscious sedation. A 6-F intermediate catheter (Navien, ev3 Neurovascular, Irvine, CA, USA) was placed in the internal carotid artery through a femoral sheath. The Solumbra technique was commonly performed with the use of a Solitaire FR device (Medtronic, Irvine, CA, USA). Aspiration through a 5- or 6-F intermediate intracranial catheter (Penumbra, Alameda, CA, USA) was conducted if necessary. Blood flow recovery was evaluated after each thrombectomy. For residual stenosis in cases with *in situ* thrombosis, balloon angioplasty and stent placement were performed at the discretion of the operator. Intra-arterial thrombolysis or intra-catheter tirofiban administration might have also been performed as rescue therapies.

### Data collection and measurements

Collected data included general characteristics, baseline National Institutes of Health Stroke Scale (NIHSS) score, stroke onset to door time (ODT), stroke onset to puncture time (OPT), door to puncture time (DPT), ORT and symptomatic intracranial hemorrhage (sICH). Successful reperfusion was defined as modified thrombolysis in cerebral infarction scale (mTICI) 2b-3. The mRS was determined preferentially by an in person follow-up appointment or *via* structured phone interview at 90 days.

### Statistical analysis

Dichotomous data were summarized as absolute values and percentages. Continuous variables are presented as median values and interquartile ranges (IQRs). Univariate comparisons between groups were performed using Mann–Whitney U tests, Chi-square tests, and Fisher’s exact tests, as appropriate. Multivariable logistic regression analysis for predictors of good clinical outcomes was performed for variables significant at the *P* < 0.1 level in univariate analyses (i.e., the Enter method). Odds ratios (ORs) and their 95% confidence intervals (CIs) were calculated. SPSS 26.0 (IBM, Armonk, NY) was used for analysis, and a *P*-value < 0.05 was considered statistically significant.

## Results

Forty patients who underwent EVT due to LVO in the anterior circulation met the inclusion criteria. Table 1 indicates the baseline characteristics, procedural and clinical variables and outcomes at 90 days. Of the overall 40 patients, good outcome was achieved in 11 (27.5%) patients. The median age was 71 (62–80) years and 75.0% of the patients were male. Median baseline NIHSS score was 20. Atrial fibrillation, hypertension, diabetes mellitus, and smoking were found in 14 (35.0%), 25 (62.5%), 7 (17.5%), and 9 (22.5%) patients, respectively. IV alteplase was performed before EVT in 12 (30.0%) patients. The median ischemic core volume (CBF < 30%) and mismatch volume determined by RAPID software were 91 (80–104) ml and 111 (88–162) ml, respectively. The median SIV was 6.8 (3.7–9.2) ml, and the median CIV was 82.5 (75.2–95.5) ml. Thirty-four (85.0%) patients had mismatch ratio ≥ 1.8. The median HIR was 0.7 (0.6–0.8) and the median ASPECTS was 4. CTA revealed the range of collaterals [Grade 4, *n* = 0 (0%); 3, *n* = 3 (7.5%); 2, *n* = 7 (17.5%); 1, *n* = 17 (42.5%); 0, *n* = 13 (32.5%)]. The mean OIT, OPT, and ORT were 233 (104–282), 277 (168–343), and 356 (294–439) min, respectively. Twenty-one (52.5%) patients had occlusions in M1 and 19 (47.5%) patients had occlusions in ICA. Successful recanalization after EVT was achieved in 37 (92.5%) patients. ICH and sICH were observed among 15 (37.5%) and 6 (15.0%) patients.

**TABLE 1:** Baseline, procedural, and outcome parameters.

Characteristics	All (*n* = 40)	Good (*n* = 11)	Poor (*n* = 29)	*P*
Age	71 (62–80)	65 (62–69)	75 (63–82)	0.030
Male gender (%)	30 (75.0)	10 (90.9)	20 (69.0)	0.233
NIHSS score	20 (15–27)	15 (11–23)	20 (17–31)	0.109
**Clinical history (%)**				
Atrial fibrillation	14 (35.0)	3 (27.3)	11 (37.9)	0.715
Hypertension	25 (62.5)	7 (63.6)	18 (62.1)	0.999
Diabetes mellitus	7 (17.5)	2 (18.2)	5 (17.2)	0.999
Smoking	9 (22.5)	3 (27.3)	6 (20.7)	0.686
IV alteplase (%)	12 (30.0)	4 (36.4)	8 (27.6)	0.704
Ischemic core ml	91 (80–104)	87 (81–99)	93 (80–107)	0.308
SIV ml	6.8 (3.7–9.2)	4.3 (2.1–5.5)	7.8 (4.5–11.0)	0.021
CIV ml	82.5 (75.2–95.5)	79.9 (76.2–94.6)	84.3 (74.1–97.4)	0.811
Mismatch ml	111 (88–162)	160 (103–185)	103 (71–139)	0.028
Mismatch ratio (%)				0.162
≥ 1.8	34 (85.0)	11 (100.0)	23 (79.3)	
< 1.8	6 (15.0)	0 (0)	6 (20.7)	
HIR	0.7 (0.6–0.8)	0.7 (0.5–0.8)	0.7 (0.6–0.8)	0.437
ASPECTS	4 (2–5)	5 (4–5)	3 (2–5)	0.064
Collateral score				0.222
0	13 (32.5)	2 (18.2)	11 (37.9)	
1	17 (42.5)	6 (54.5)	11 (37.9)	
2	7 (17.5)	1 (9.1)	6 (20.7)	
3	3 (7.5)	2 (18.2)	1 (3.4)	
4	0 (0)	0 (0)	0 (0)	
OIT min	233 (104–282)	163 (94–269)	242 (106–287)	0.402
OPT min	277 (168–343)	245 (193–319)	302 (160–345)	0.676
ORT min	356 (294–439)	319 (292–400)	376 (295–466)	0.148
**Lesion site (%)**				
M1	21 (52.5)	6 (54.5)	15 (51.7)	0.873
ICA	19 (47.5)	5 (45.5)	14 (48.3)	
mTICI 2b-3 (%)	37 (92.5)	11 (100.0)	26 (89.7)	0.548
Any ICH (%)	15 (37.5)	3 (27.3)	12 (41.4)	0.486
sICH (%)	6 (15.0)	1 (9.1)	5 (17.2)	0.999

**FIGURE 3:**
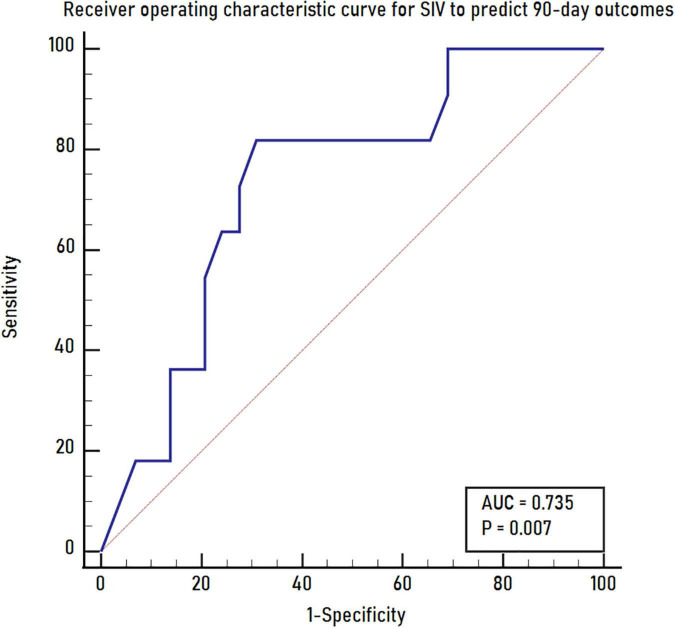
The receiver operating characteristics curve for SIV to predict 90-day outcomes. SIV, subcortical ischemic volume.

In univariate analysis, younger age (*P* = 0.030), smaller SIV (*P* = 0.021), and larger mismatch volume (*P* = 0.028) were noted in the good outcome group than in the poor outcome group. The ASPECTS was 5 and 3 for the good and poor outcome groups, respectively (*P* = 0.064). No significant differences were noted between the 2 groups regarding sex, baseline NIHSS, clinical history, CIV, HIR, CTA collateral score, treatment with IV alteplase, OIT, OPT, ORT, occlusion sites, successful recanalization, ICH and sICH (all *P* > 0.1). Further multivariate logistic regression analysis showed that only a smaller SIV (OR 0.801; 95% CI 0.644–0.996; *P* = 0.046) was an independent predictor for good outcomes at 90 days (Table 2). The receiver operating characteristic curve indicated a moderate value of SIV for predicting good outcomes, with an area under the receiver operating characteristics curve of 0.735 (95% CI 0.572–0.862; *P* = 0.007) (Figure 3). In the receiver operating characteristic curve, the optimal cut-off value of SIV for predicting good outcome was ≤ 5.5 ml with a sensitivity of 82% and specificity of 69%.

**TABLE 2:** Multivariable logistic regression analysis for predictors of outcomes at 90 days.

	β-coefficient	OR (95% CI)	*P*-value
Age	−0.066	0.936 (0.870–1.007)	0.077
SIV	−0.222	0.801 (0.644–0.996)	0.046
Mismatch	0.006	1.006 (0.996–1.016)	0.276
ASPECTS	0.297	1.346 (0.913–1.984)	0.133

Variables entered: age, SIV, mismatch, ASPECTS.

## Discussion

In this study, we compared the clinical and imaging features of AIS patients receiving CTP with an ischemic core > 70 ml within 6 h of stroke onset or time last known well to identify predictors of 90-day outcomes after EVT. We found that CTP-based SIV was independently associated with clinical outcomes.

A large core was associated with poor outcome in patients with LVO and has been listed as an exclusion criterion in many studies. In this study, we observed a good outcome in 27.5% of the patients, which was comparable with previous literature (Kerleroux et al., 2020). Other studies reported a higher rate of good outcomes. Rebello et al. (2017) reported that 40% (4/10) of patients treated with EVT achieved functional independence at 90 days in a small matched case-control study. But the cases they reported were younger than our patients. Yoshimoto et al. (2021) found that 42.9% (21/49) of patients achieved 90-day good functional outcomes in the EVT group. We think that the rapid ORT might be positive for this highest rate of good outcome in their series [234 (160–424) min in Group A: 70–100 ml; 213 (201–373) min in Group B: 101–130 ml; 154 (117–198) min in Group C: > 130 ml]. Therefore, although it was rare, previous studies suggested that young and short ORT patients might have incomplete cytotoxic edema unrelated to core volume measurement, and their functional outcomes may be preserved after complete reperfusion.

Previous studies have examined the association between the infarct region and functional outcome at 90 days. One important difference from our study is the treatment strategy provided to the patient population. Most patients in prior studies were either treated with intravenous thrombolysis or managed conservatively. Only Xing et al. (2021) included patients who underwent EVT and used the ASPECTS as the assessment method. Our study included patients undergoing CTP, which provided a more accurate estimation of both infarct region and core volumes. In this study, only small baseline SIV was found to be an independently predictive factor of good outcome. This result might be associated with individual regions covering different amounts of brain tissue with different functions. Although the mRS scale is the most widely used tool to evaluate the prognosis of stroke, it is mainly determined by motor disability, particularly walking. The territory of the brain associated with persistent motor disability was clustered in an area centered in the deep periventricular white matter and in the adjacent internal capsule (Cuingnet et al., 2011). Reorganization at the subcortical level remains insufficient for the patient’s recovery in case of damage to the main motor outflow tract, finally leading to a lower likelihood of paralysis recovery. Moreover, an analysis of HERMES (Highly Effective Reperfusion Using Multiple Endovascular Devices) pointed that only 12% of the relationship between the EVT effect and functional outcomes was mediated by infarct volume (Boers et al., 2019). Ernst et al. (2015, 2018) reported the significant differences in functional outcome among brain regions after correcting for infarct volume, suggesting that ischemic core volume was a useful marker of functional outcome but should be considered in the context of lesion location. Most previous EVT trials set baseline ischemic core < 70 ml as the study exclusion criterion in an attempt to exclude patients with large ischemic core infarcts that were assumed to be unlikely to benefit from EVT. This result may raise a concern that a single volumetric selection criterion might have negative results in determining patients eligible for EVT. Our study suggested that patients who were considered ineligible for EVT because of large core but showed small SIV could still benefit from EVT (Figures 2B, C).

Age and mismatch volume were correlated with good prognosis in a univariate analysis. Age is considered to be an independent predictor of outcome in acute ischemic stroke due to LVO treated with EVT (Castonguay et al., 2014). Gilgen et al. (2015) reported that patient age was an independent predictor of outcome in patients with diffusion-weighted magnetic resonance imaging (DWI) lesions > 70 ml. EVT is effective in elderly patients, but the likelihood of achieving a good outcome reduces as age increases (Alawieh et al., 2019). Compared with younger patients, elderly stroke patients stay longer in the hospital, are less often discharged home, and their mortality is higher (Saposnik et al., 2008). While selecting patients, most centers mainly considered the volume of the ischemic core, while only a few centers considered the volume of mismatch. Within FRAME trail, Olivot et al. (2021) found that mismatch profile influenced outcomes after EVT and reported that about 29% of the AIS patients in early window did not have target mismatch, and target mismatch profile were independently associated with a higher rate of functional recovery after EVT. However, the CLEAR (Nguyen et al., 2022) study implied that it was not necessary to consider the penumbra at all, studies that directly compare ischemic core and penumbra parameters are needed to prove this finding.

Endovascular treatment is often not performed in patients with a large ischemic core because of an increased risk of hemorrhagic complications. However, a prior meta-analysis showed that, compared with standard medical treatment, EVT did not increase the odds of sICH in these patients (Sarraj et al., 2020). The rate of sICH in our cohort was 15.0% (6/40) and was not statistically significant in the two groups, which suggested EVT is a safe treatment for patients with large infarct volumes less than 130 ml.

Our study has some limitations. First, this study was a retrospective study that likely resulted in selection bias. Second, the sample size was small, which may have exaggerated the difference between the two groups. The results may not be generalizable and should be interpreted with caution. Third, CTP volumes, SIV and CIV were calculated using two different software platforms, RAPID and ITK-Snap, which can lead to heterogeneity of the data. Finally, the accuracy of manual infarct segmentation is limited because it is often difficult to delineate the boundaries. Finally, the manual infarct segmentation could be affected by unertainties and/or imprecisions because it is often difficult to delineate the boundaries. Further study should focus on implement image preprocessing based on fuzzy logic as scientific literature suggests (Khalid et al., 2014; Zhang, 2014; Versaci et al., 2015).

In conclusion, AIS patients with a large core may benefit from EVT. A smaller baseline SIV is a positive variable for predicting good outcomes in this subgroup, and we favor baseline SIV as an important variable for making treatment decisions.

## Data availability statement

The raw data supporting the conclusions of this article will be made available by the authors, without undue reservation.

## Ethics statement

The studies involving human participants were reviewed and approved by the Ethics Committee of The First Affiliated Hospital with Nanjing Medical University. Written informed consent for participation was not required for this study in accordance with the national legislation and the institutional requirements.

## Author contributions

SL, YL, and YD: concepts and study design. SL and YG: data analysis and interpretation. YG and YH: data collection. YG, LZ, and ZJ: manuscript drafting. YG and YC: statistical analysis. All authors read and approved the final manuscript.

## Footnotes

1
http://www.itksnap.org
