# Corrigendum: Smaller baseline subcortical infarct volume predicts good outcomes in patients with a large core in early acute ischemic stroke after endovascular treatment

**DOI:** 10.3389/fnins.2023.1160678

**Published:** 2023-03-03

**Authors:** Yiming Gu, Yasuo Ding, Yu Hang, Yuezhou Cao, Zhenyu Jia, Linbo Zhao, Ying Liu, Sheng Liu

**Affiliations:** ^1^Department of Interventional Radiology, The First Affiliated Hospital of Nanjing Medical University, Nanjing, China; ^2^Department of Interventional Radiology, Suzhou Municipal Hospital Affiliated to Nanjing Medical University, Suzhou, China; ^3^Department of Neurosurgery, Taizhou People's Hospital, Taizhou, China; ^4^Department of Neurology, Taizhou People's Hospital, Taizhou, China

**Keywords:** acute ischemic stroke, endovascular thrombectomy, infarct volume, predictor, good outcomes

In the published article, there was an error. [References are cited in the Results section].

A correction has been made to **[**Results**]**, [Paragraph 1 and 2]. This sentence previously stated:

“[1) Median baseline NIHSS score was 20 (Saposnik et al., 2008; Castonguay et al., 2014; Khalid et al., 2014; Zhang, 2014; Ernst et al., 2015, 2018; Gilgen et al., 2015; Versaci et al., 2015; Alawieh et al., 2019; Boers et al., 2019; Sarraj et al., 2020; Olivot et al., 2021; Nguyen et al., 2022).

2)The median HIR was 0.7 (0.6–0.8) and the median ASPECTS was 4 (Campbell et al., 2015; Albers et al., 2018; Demeestere et al., 2018; Nogueira et al., 2018).

3) The ASPECTS was 5 (Campbell et al., 2015; Albers et al., 2018) and 3 (Campbell et al., 2015; Albers et al., 2018; Demeestere et al., 2018; Nogueira et al., 2018) for the good and poor outcome groups, respectively (*P* = 0.064).]”

The corrected sentence appears below:

“[1) Median baseline NIHSS score was 20.

2) The median HIR was 0.7 (0.6–0.8) and the median ASPECTS was 4.

3) The ASPECTS was 5 and 3 for the good and poor outcome groups, respectively (*P* = 0.064).]”

The authors apologize for this error and state that this does not change the scientific conclusions of the article in any way. The original article has been updated.

